# Brain metastases from non-small cell lung cancer: molecular subtypes and emerging CNS-directed precision therapies

**DOI:** 10.3389/fonc.2026.1717432

**Published:** 2026-01-22

**Authors:** Mehek Sharma, Anvay Shah, Kimberly A. Rivera-Caraballo, Girindra Raval, Balveen Kaur, Gerald C. Wallace

**Affiliations:** 1 Medical College of Georgia at Augusta University, Augusta, GA, United States; 2 Georgia Cancer Center at Wellstar Health, Augusta, GA, United States; 3 Department of Pathology, Medical College of Georgia and Georgia Cancer Center at Augusta University, Augusta, GA, United States

**Keywords:** BBB penetration, brain metastases, molecular biomarkers, non-small cell lung cancer, patient-centered outcomes, targeted therapies

## Abstract

Non-small-cell lung cancer (NSCLC) is a leading cause of morbidity and mortality globally, due in large part to the development of NSCLC-associated brain metastases (L-BM). Upon initial presentation, 11-26% of patients with NSCLC will have L-BM, while half of patients with NSCLC will develop L-BM over the course of their disease. The emergence of PD-1/PD-L1 immunotherapy and targeted therapies for *EGFR*, *ALK*, and *ROS1* mutations has transformed the treatment landscape and improved outcomes for select patient populations. CNS progression remains a major challenge due to therapy resistance, the blood–brain barrier (BBB), and the unique molecular and transcriptomic adaptations exhibited by NSCLC brain metastases which differs markedly from primary lung tumors. In this review, we examine the molecular drivers of CNS metastasis, oncogenic signaling-targeted therapies, and next-generation CNS drug-delivery strategies including intraventricular or intranasal administration, focused ultrasound, nanocarriers, and efflux transporter modulation. Furthermore, we provide a comprehensive update on recent and ongoing preclinical and clinical studies, highlighting novel CNS-penetrant agents with demonstrated intracranial efficacy. Understanding these mechanisms and refining targeted approaches are critical to improving CNS disease control, survival outcomes, and quality of life for NSCLC patients with brain involvement.

## Introduction

Lung cancer is the second most common malignancy in the United States. Lung cancers are broadly divided into small cell and non-small cell lung cancer (SCLC and NSCLC, respectively). SCLC is a neuroendocrine carcinoma with a poor prognosis (1, 2), while NSCLC constitutes 85% of all other lung cancer diagnoses (3). NSCLC tumors are further divided by pathologic subtypes: adenocarcinoma, squamous cell carcinoma, or large cell carcinoma (4). Brain metastases from NSCLC (L-BM) occur in up to 50% of all patients, with likely increasing incidence as patients survive with high-stage disease (5). Survival with L-BM has significantly improved over time, with recent reports indicating a median survival of 12–24 months (6, 7). The importance of recognizing and managing L-BM has increased recently, since patients without stage IV disease live longer with increasing year-over-year risk for developing L-BM (1, 7).

Historically, management of L-BM relied on radiation and chemotherapy, but the advent of targeted therapies and immunotherapies has transformed the therapeutic landscape, demonstrating improved central nervous system (CNS) penetration and efficacy. Despite these advances, our understanding of how specific molecular subtypes influence the development, progression, and treatment of brain metastases in NSCLC patients is yet to be elucidated. Synthesizing existing knowledge linking NSCLC molecular subtypes to CNS metastatic risk, resistance mechanisms, and therapeutic response, is crucial to inform surveillance protocols, treatment selection, and clinical trial design. In this review, we will provide an overview of NSCLC development and molecular characteristics, as well as the current treatment strategies to improve outcomes for patients with L-BM.

## Etiology and statistics

The American Cancer Society (ACS) projects more than 226,650 new cases of lung cancer and 124,730 deaths in the United States in 2025 (8). The median age at diagnosis is 71 years with 90% of diagnoses and deaths occurring in patients older than 55 years (9, 10). Lung cancer accounts for the highest number of cancer-related deaths, with a lifetime probability of 6.2% in men and 5.8% in women and an incidence of 64.1 cases per 100,000 in men compared to 50.3 per 100,000 in women (8). Incidence varies by race and sex, where Black men have the highest incidence and Hispanic women having the lowest. Cigarette smoking remains the most common risk factor with 80% of NSCLC patients admitting a history of smoking, while environmental radon exposure marks a distant second place (8, 11).

The one, three, and five-year survival rates following diagnosis of L-BM due to any lung cancer are 24%, 6%, and 3%, respectively (12). NSCLC accounts for approximately 25% of all brain metastases (13) and studies have shown 40% prevalence of L-BM (1, 14). L-BM contributes to one in three deaths from NSCLC and is a key reason why lung cancer is the leading cause of death worldwide (6, 8, 15). The association with L-BM is highest for non-squamous adenocarcinoma (16–20).

Risk factors for L-BM include female sex, younger age (particularly those < 30 years old), stage at diagnosis, larger primary tumor size, lymph node involvement, and adenocarcinoma phenotype (16, 18–21). Ceresoli and colleagues examined 112 patients with locally advanced NSCLC and found that L-BM were more common in patients <60 years old and in patients with bulky mediastinal lymph nodes, which they defined as greater than two centimeters in size (17). Bajard and colleagues showed similar findings, along with a particularly high risk among patients < 30 years (22).

At diagnosis, approximately 26% of patients with stage IV NSCLC had L-BM (23). While L-BM can define stage IV NSCLC disease, patients with stage I-III disease are not necessarily spared for a future L-BM diagnosis, with reported incidences of 25.6% for stage I/II and 19.3% for stage III disease (24). The cumulative five-year risk of L-BM increases with stage, ranging from 3.6% in stage I to 25% in stage III disease (25). Moreover, two relatively large cohort studies from the early 2000’s found a statistically significant correlation between the prevalence of L-BM over time if the primary tumor was large and lymph node staging was higher (22, 26). For this reason, patients with stage III or stage IV disease are recommended to receive a screening contrasted MRI of the brain at first diagnosis.

## Transcriptomic landscapes of NSCLC brain metastases

Recent genomic and spatial transcriptomic studies have revealed that brain metastases arising from NSCLC frequently exhibit distinct molecular signatures compared to primary lung tumors and extracerebral metastatic sites. Brain metastases have been shown to demonstrate higher chromosomal instability and increased copy number variations (CNVs), particularly involving tumor suppressor loci such as *CDKN2A/B*, compared to primary tumors (34% vs. 13%, respectively) (27, 28). Spatial transcriptomic profiling of NSCLC primary and brain metastatic regions indicates that brain metastasis niches are characterized by upregulation of extracellular matrix (ECM) and cancer−associated fibroblast signatures, with gene otology (GO) analyses highlighting collagen–ECM–mediated signaling and fibrosis (29). Comparative expression analyses further reveal increased expression of metabolic pathways such as oxidative phosphorylation, glycolysis, fatty−acid metabolism, hypoxia responses, angiogenesis, and *PI3K/AKT/mTOR* signaling in brain metastases relative to earlier−stage primary tumors, which is consistent with metabolic reprogramming to support survival in the CNS microenvironment (30). Brain metastatic cells also display transcriptional adaptations that reflect close interaction with neural and glial components. Multi−cancer brain metastasis studies suggest that tumor cells in the brain partially adopt neural−like phenotypes to thrive intracranially, an adaptation less evident in non−CNS metastatic sites (29, 31). Together, these data suggest that CNS lesions harbor a unique immune and metabolic landscape not observed in metastatic lesions in other organs (ex. liver, bone, or other extracerebral metastases).

## Genetic drivers of NSCLC and L-BM prognosis

While NSCLC brain metastases demonstrate a broad spectrum of unique genomic and transcriptomic alterations, a subset of well-characterized molecular subtypes remain central to understanding the clinical behavior, therapeutic vulnerabilities, and prognosis of CNS disease in lung cancer. Among the principal oncogenic drivers of NSCLC, mutations in *KRAS* and *EGFR* as well as rearrangements of *ALK* and *ROS1* are the most prevalent (4). Among all subtypes, patients with *EGFR-* and *ALK-*driven L-BM have a more favorable prognosis in the context of CNS-penetrant targeted therapies (32).

### *KRAS* mutated

The *KRAS* gene is the most commonly mutated oncogene in NSCLC, accounting for nearly 30% of all cases (33, 34). KRAS proteins are activated by growth factors (ex. EGF, PDGF, FGF), chemokines, Ca2+, or receptor tyrosine kinases (RTKs) and can activate downstream effector pathways such as the RAF- MEK- ERK signaling pathway and *PI3K- AKT* pathway to promote cell growth and proliferation (34, 35). About 90% of *KRAS* mutations are found in codon 12, with G12C mutations being most prevalent (39-42%), followed by G12V mutations (19%) and G12D (17%) (33, 36). Distributions of *KRAS* mutations have been well-documented and shown to vary among race, sex, and smoking status. Specifically, *KRAS* mutations are linked to Caucasian ethnicity (26% in Caucasians vs. 11% in Asians), female sex (31.35% in females vs. 23.7% in males), adenocarcinoma histology (37.2% in adenocarcinomas vs. 4.4% in squamous cell carcinomas), and a history of smoking, where G12C mutations had the highest rate of current smokers at 43% and G12D mutations were common among never or light smokers (33, 36). The *KRAS* mutation frequently coexists with changes in other significant cancer-related pathways, including *TP53, STK11*, and *KEAP1* in 10.3–28.0%, 6.3–23.0%, and 17.8–50.0% of patients, respectively (37). Importantly, a large cohort study of 899 patients with stage IV NSCLC found that there was no significant difference in the incidence of brain metastases in patients with the G12C mutation than those without (37.2% vs. 33.5%, p=0.26), suggesting that brain metastases are common in *KRAS*-mutant NSCLC regardless of *KRAS* subtype, and concurrent mutations in *TP53, STK11*, and *KEAP1* may further exacerbate the treatment resistance observed in these patients (38).

The KRAS protein has been historically deemed as “undruggable”; however, recent advancements in targeted therapies have resulted in the successful development of two drugs in 2022: sotorasib and adagrasib. By binding to the KRAS G12C cysteine residue and locking the abnormal receptor in an inactive conformation, these drugs prevent oncogenic signaling and cell proliferation, leading to potent tumor regression (39).

The CodeBreaK 200 trial, published in 2023, was the first Phase III randomized trial to demonstrate improved outcomes for a KRAS G12C inhibitor. Sotorasib demonstrated superior progression-free survival (PFS) versus docetaxel (mitotic inhibitor), and importantly, intracranial efficacy was observed in a subset of patients (33.3% vs. 15.4%), despite the exclusion of those with active brain metastases at enrollment. Similarly, adagrasib also achieved significant CNS penetration and efficacy. In patients with untreated, asymptomatic brain metastases, adagrasib demonstrated promising CNS penetration, as evidenced by an objective response rate (ORR) of 42% in the KRYSTAL-1 trial published in 2023 (40). The KRYSTAL-12 trial, part of ongoing KRYSTAL-2 program, presented in 2023, has confirmed PFS and ORR benefits over docetaxel in *KRAS* G12C-mutant NSCLC (see **Table 1**). The ongoing KRYSTAL-7 trial is further investigating adagrasib in combination with checkpoint inhibitors such as pembrolizumab. The Phase II cohort demonstrated that PD-L1 positive patients achieved a tumor proportion score (TPS) ≥50%, and the combination achieved an ORR of 63% and a disease control rate of 84%, supporting the potential benefit of combining adagrasib with checkpoint inhibitors to improve outcomes (41). See **Table 1** for key trial efficacy data.

**Table 1:** A comparison of key NSCLC clinical trials by mutation, treatment, and efficacy outcomes.

Trial/Study	Status/Year	Mutation	Treatment	Intracranial ORR	Systemic ORR	PFS (mo)	OS (mo)	DoR (mo)	Clinical Implications
FLAURA2 (NCT04035486)	Completed/2023	EGFR (ex19del, L858R)	Osimertinib + platinum-pemetrexed vs osimertinib	Not reported separately	83% vs 76%	25.5 vs 16.7	NR vs 36.7	24.0 vs 15.3	Durable CNS benefit; combo improves depth and durability of response
HERTHENA-Lung01 (Phase II) (NCT04619004)	Ongoing/2021–2026	EGFR	Patritumab deruxtecan (HER3-DXd)	33.3% (10/30)	29.80%	5.5	11.9	6.4	Post-EGFR-TKI cohort, included untreated brain mets
MARIPOS (NCT04487080)	Completed/final data published 2025	EGFR (ex19del, L858R)	Amivantamab + Lazertinib vs Osimertinib	78% vs 77%	86% vs 85%	23.7 vs 16.6	NR vs 36.7	25.8 vs 16.8	First-line chemotherapy-free regimen; improvement in PFS and OS
SAVANNAH (Phase II) (NCT03778229)	Ongoing	EGFR ex19del/L858R + MET amp	Savolitinib plus osimertinib	Not Specified	56.30%	7.4	Not specified	7.1	Dual EGFR + MET inhibition overcomes MET-driven osimertinib resistance
TROPION-Lung01 (Phase III) (NCT04656652)	Completed/2024	NSCLC (non-squamous, PD-L1 stratified)	Datopotamab deruxtecan vs docetaxel	NR	26.4% vs 12.8%	4.4 vs 3.7	14.6 vs 12.3	7.1 vs 4.4	Some CNS inclusion; not powered for intracranial outcomes
TROPION-Lung05 (Phase II) (NCT04484142)	Ongoing/2022-2025	EGFRm NSCLC post-EGFR-TKI	Datopotamab deruxtecan	Not specified	43.6%	5.4	18.3	7.0	Single-arm EGFRm NSCLC cohort. Broad NSCLC response benefits
CodeBreaK 200 (Phase III) (NCT04303780)	Completed enrollment/data available by 2023-2024	KRAS G12C	Sotorasib vs docetaxel	33.3% vs 15.4%	28.1% vs 13.2%	5.6 vs 4.5	10.6 vs 11.3	8.6 vs 6.8	Sotorasib outperformed docetaxel; brain mets excluded but IC efficacy seen
KRYSTAL-12 (Phase III) (NCT04685135)	Completed/2024	KRAS G12C	Adagrasib vs docetaxel	24% overall; 40% in CNS pts	32% vs 9%	5.5 vs 3.8	NR (immature)	NR	Ongoing phase III; Significant systemic and CNS responses
KRYSTAL-1 (CNS cohort) (NCT03785249)	Ongoing/preliminary results reported 2023	KRAS G12C	Adagrasib	42%	30%	5.4	11.4	11.2	Dedicated CNS cohort, untreated brain mets. First demonstration of CNS activity
PROFILE 1014 (Phase III) (NCT01154140)	Completed/2014	ALK	Crizotinib vs chemotherapy	NR	74% vs 45%	10.9 vs 7.0	NR (crossover confounding)	11.3 vs 5.8	Landmark 1L ALK trial. Limited CNS penetration; modest control
CROWN (Phase III) (NCT03052608)	Completed/2024	ALK	Lorlatinib vs crizotinib	66% vs 20%	78% vs 22% (brain mets); 78% vs 45% (no brain mets)	NR vs 7.2	NR vs 11.0	NR	Significant CNS efficacy advantage
ALEX (Phase III) (NCT02075840)	Completed/2017-2018	ALK	Alectinib vs crizotinib	78.6% vs 40.0% (among those with measurable/non-measurable CNS metastases without RT)	82.9% vs 75.5% (overall population)	34.8 vs 10.9	NR	NR vs 11.1 (among those with measurable/non-measurable CNS metastases without RT)	Alectinib delayed CNS progression; became frontline ALK+ NSCLC therapy
ALTA-1L (NCT02737501)	Completed/2021	ALK	Brigatinib vs crizotinib	78% vs 26% among those with measurable brain metastases	74% vs 62%	48% vs 15% at 2 years among those with measurable brain metastases	76% vs 74% at 2 years overall	NR vs 14.8	Brigatinib showed superior systemic and intracranial efficacy

ORR, Objective Response Rate; PFS, Progression Free Survival; OS, Overall Survival; DoR, Duration of Response; NR, Not Reached; EGFR, Epidermal Growth Factor Receptor; TKI, Tyrosine Kinase Inhibitor; ALK, Anaplastic Lymphoma Kinase; cFAS, CNS full analysis set; cEFR, CNS evaluable-for-response set; RT, radiotherapy.

### *EGFR* mutated

Epidermal growth factor receptor (EGFR) a transmembrane tyrosine kinase of the ErbB/HER family, homodimerizes and autophosphorylates to activate pro-tumorigenic Ras, PI3K, and JAK pathways (42, 43). *EGFR* mutations in NSCLC are more common in East Asians, women, and nonsmokers (44–46), and are associated with a higher risk of CNS metastasis (47). Both the L858R point mutation and exon 19 deletion subtypes of *EGFR* mutations exhibit a significantly higher risk of L-BM, with a comparable incidence observed between the two (39.5% vs. 34.5%, p=0.770) (48). Multiple studies have shown an association with *EGFR* mutation status and L-BM based on circulating tumor DNA in serum and cerebrospinal fluid (CSF) (49, 50). *EGFR* mutations in cerebrospinal fluid were found in 57% of patients with L-BM, and in 81% of these patients who had leptomeningeal disease (LMD), suggesting diagnostic and prognostic purposes in this space (51, 52).

From the United States, a meta-analysis of 68 studies shows an *EGFR* mutation prevalence of 24% with significant impact on life expectancy (53, 54). A large-scale Chinese data analysis showed higher *EGFR* mutation rates among stage IA than in stages IIB and IIIA (55). Patients with early-stage NSCLC and *EGFR* mutations had shorter disease-free survival, an increased risk of disease recurrence, lymphovascular invasion, intrapulmonary metastasis, lymph node involvement, and distant metastasis in early-stage lung cancer (46). Cumulative incidence of L-BM is likely 40% higher among *EGFR* mutated NSCLC and is associated with worse survival (56).

Three generations of EGFR-TKIs have been approved for NSCLC patients (57). First-generation (gefitinib, erlotinib, and icotinib) and second-generation (afatinib and dacomitinib) irreversible small molecule inhibitors have exhibited robust and durable responses in patients with Ex19del and L858R mutations (57). However, most patients eventually develop acquired resistance to EGFR targeted therapy within 9–14 months, with the T790M mutation most commonly found (58, 59). Most recurrences post-use of first- and second-generation EGFR inhibitors are in the CNS since they do not have CNS efficacy. Fortunately, osimertinib, an irreversible third-generation EGFR inhibitor, effectively overcomes the T790M resistance mutations while also offering higher selectivity, lower toxicity, and improved blood-brain barrier (BBB) penetration (57). The FLAURA trial demonstrated that first-line osimertinib significantly improved median PFS and with lower rates of adverse effects among patients with L-BM compared to earlier generation EGFR-TKIs (60). Moreover, these benefits were sustained regardless of T790M status (61, 62), because of CNS penetration of Osimertinib (63). However, if given as first line treatment and dose is de-escalated to reduce adverse events, osimertinib can promote L-BM or exacerbate the incidence if already an L-BM patient (64).

Combination strategies have further improved CNS control and survival outcomes in *EGFR*-mutated lung cancer. The FLAURA2 study, published in 2023, showed that the combination of osimertinib and chemotherapy improved PFS and OS compared to osimertinib monotherapy in stage IV *EGFR*-mutated lung cancer (65). Subsequently, in the MARIPOSA study using amivantamab + lazertinib has shown similarly improved efficacy in patients with CNS metastasis compared to osimertinib alone, including a significantly prolonged PFS, OS, and DOR while maintaining intracranial objective response rates in patients that harbored the exon 19 deletion or exon 21 L858R substitution mutation (66). This led to amivantanab + lazertinib being recognized as a new first-line treatment for *EGFR*-mutated NSCLC. More recently, patritumab deruxtecan (HER3-DXd), the first HER3-directed antibody drug conjugated with deruxtecan payload designed to minimize systemic toxicity, demonstrated notable CNS activity in patients with EGFR-mutated non-small cell lung cancer with brain metastases. In this phase II trial (HERTHENA-Lung01), an intracranial ORR of 33.3% and a CNS DOR of 8.4 months was observed, opening a new HER-3 mediated therapeutic avenue for EGFR-mutated NSCLC patients (67). A pooled analysis of the TROPION-Lung05 (phase 2) and TROPION-Lung01 (phase 3) trials has shown that after progression on osimertinib, the datopotamab-deruxtecan combination (Dato-DXd) exhibits encouraging and durable antitumor activity (68). Most recently, early data from the SAVANNAH II, trial demonstrated that the combination of osimertinib plus savolitinib resulted in clinically meaningful and durable efficacy for MET-mediated resistance with CNS efficacy in NSCLC (69).

Additionally, multiple trials have shown efficacy of combinatorial treatments utilizing EGFR-TKI’s, chemotherapy, and immunotherapy (43). For example, EGFR-TKI and VEGF-inhibitors have been shown to delay T790M-mediated resistance compared with single-agent EGFR-TKI and resulted in tumor regression in L858R/T790M-positive tumors (43). All of these studies represent recent advances (2023-2025) in the evolving treatment landscape for *EGFR*-mutated NSCLC, demonstrating a meaningful shift toward molecular targeted therapies to address CNS involvement. Detailed efficacy data from these trials are summarized in **Table 1**.

### *ALK* rearranged

Anaplastic lymphoma kinase (ALK) is a receptor tyrosine kinase in the insulin receptor family. Homodimerization of ALK activates multiple cell proliferation pathways, including PLC-gamma, JAK-STAT, and mTOR (70). *ALK* rearrangements occur in approximately 5% of NSCLC cases and result in constitutively active fusion proteins that drive tumor growth (71, 72). In NSCLC, the most prevalent *ALK* rearrangements include the echinoderm microtubule-associated protein like 4 (*EML4*) fusion with *ALK* (*EML4-ALK*) resulting from a paracentric inversion on chromosome 2p. The EML4-ALK fusion kinase has been implicated in tumorigenicity and driving oncogenesis in up to 6.7% of NSCLC and is commonly seen in Asian patients under the age of 50, with a light-smoking or non-smoking history (73, 74).

Clinically, pathogenic *ALK* rearrangements are not only significant in guiding treatment options but also in their association with brain metastases (75–77). *ALK* rearranged disease harbors a 23.8% risk of L-BM at initial diagnosis and nearly 60% incidence of L-BM after three years (78). Multiple other studies have corroborated these findings, reporting *ALK*-positivity in 30-40% of patients with L-BM at time of diagnosis (51, 79–83). A study of 131 ALK-positive NSCLC patients found that those with *ALK* variant 1 (*EML4-ALK* fusion) had larger brain metastases compared to non-variant 1 patients (median size 16.89 mm vs. 11.01 mm, P = 0.031) and significantly shorter time to treatment failure than non-variant 1 with baseline L-BM when treated with first-line crizotinib (median TTF: 9.1 vs. 14.9 months, HR = 2.68, P = 0.037) (84).

The therapeutic landscape of *ALK*-positive NSCLC has evolved significantly over the past decade. In the PROFILE 1014 trial completed in 2014, crizotinib, the first-generation ALK inhibitor, demonstrated systemic efficacy in *ALK*-positive NSCLC, prolonging PFS and improving systemic ORR, establishing it as a new standard for systemic treatment of the disease. However, it’s limited CNS penetration (0.26%) resulted only in modest intracranial control (74% vs. 45%). While crizotinib primarily targets the EML4-ALK fusion, resistance mutations such as L1196M, G1269A, and G1202R further reduced its clinical efficacy (85–88). These mutations affect the ATP-binding pocket of the kinase domain, altering inhibitor binding and driving therapeutic resistance, thus prompting the need for more effective CNS therapies in this patient population (89).

Subsequent ALK inhibitors markedly improved treatment options by overcoming both the CNS penetration limitations and resistance mutations of crizotinib. Alectinib achieved an intracranial ORR of approximately 79% in patients with measurable brain metastases in the ALEX trial in 2018, and brigatinib produced a similar intracranial response rate of about 78% in the ALTA-1L trial in 2021, showing strong L-BM control. Alectinib and brigatinib also overcame several crizotinib resistance mutations, yet continued to present challenges with the G1202R mutation, which confers high-level resistance in brain metastases, contributing to CNS progression and treatment failure in *ALK*-positive patients (87, 90). Importantly, lorlatinib was developed to overcome to G120R resistance, and the CROWN trial completed in 2024 demonstrates that it achieved a significant CNS efficacy advantage, with an intracranial ORR of approximately 78%, in those with brain metastases and without, along with superior PFS compared to earlier ALK inhibitors (91–93). Lorlatinib’s potency against the G120R mutation makes it a highly effective treatment option in overcoming refractory brain metastases in *ALK*-positive lung cancer (90). Key data from these trials are summarized in **Table 1**.

However, despite advances in CNS-penetrant ALK inhibitors and their high intracranial response rates, later resistance frequently arises from activation of alternative oncogenic pathways in proto-oncogenes such as *RET, EGFR, KRAS, NF1, IDH1, RIT1, ERBB2, BRAF, FGFR3, CREBBP*, and *NOTCH* (94, 95). These off-target mechanisms enable tumor cells to bypass ALK dependency and sustain tumor growth and proliferation despite continued therapy, highlighting the continued need for molecular monitoring and the development of combination therapeutic strategies to achieve durable control in *ALK*-positive NSCLC (94, 96).

### *ROS1* mutated

The *ROS1* proto-oncogene plays various roles in cellular signaling pathways associated with proliferation, cell growth, and survival. To date, there have been > 20 different ROS1 fusion proteins observed in NSCLC, with some variants more common than others. The five most common fusion with ROS1 are *CD74* (49.8%), *EZR* (23.6%), *SDC4* (9.1%), *SLC34A2* (5.1%), and *TPM3* (2.9%) (97–99).

Patients with *ROS1*-rearranged NSCLC have a common and significant risk of developing CNS metastases, although no clear increase in risk has been demonstrated specific to these fusion types (100). Several clinical factors are associated with a higher likelihood of harboring *ROS1* fusions, including female sex, younger age, and those with early diagnosis of advanced stage NSLC (101). Among 103 patients with *ROS1* mutations, 22% had L-BM at diagnosis while 30% developed L-BM later, with a median time to L-BM development of 12 months (98). Incidence of L-BM at initial diagnosis as high as 36% were reported in another study (102).

Crizotinib has achieved excellent systemic responses in treating *ROS1*-rearranged NSCLC, similar to its efficacy in *ALK*-positive disease, and was approved for *ROS1* mutant NSCLC in 2016 (103). However, its effectiveness within the CNS was again limited due to poor BBB permeability. In 2019, Patil and colleagues showed that the CNS is a common first and sole site of progression in about 47% of ROS1 NSCLC patients on crizotinib (103). This frequent CNS progression occurred after 24 months of therapy, suggesting that L-BM, despite presenting at a low percentage at the initiation of crizotinib, can occur quite late in the course of treatment, and remains a major source of morbidity (103). This limited CNS efficacy has driven the development of next-generation ROS tyrosine kinase inhibitors such as entrectinib. An integrated analysis of the ALKA-372-001, STARTRK-1, and STARTRK-2 trials from 2022 showed that entrectinib achieved an intracranial ORR of about 80%, with a median intracranial DOR of 12.9 months and median intracranial PFS of 8.8 months in patients with baseline CNS metastases (104). In patients with CNS-only progression post-crizotinib, the intracranial efficacy of entrectinib was modest, with 11% of patients achieving a partial response and 22% achieving a stable response and decrease in tumor size (104). Still, entrectinib’s CNS penetration remains superior to crizotinib, supporting entrectinib as a standard option for ROS1 fusion-positive NSCLC with L-BM (104).

## Current therapeutic approach: immunotherapy

Primary NSCLC tumors vary dramatically from L-BM lesions, although both reflect profound immunosuppressive environments. Some reports suggest that the immunosuppressive nature of primary NSCLC might contribute to L-BM (105, 106). Compared with L-BM, primary NSCLC tumors contain more lymphocytic and dendritic cell infiltrates, with no significant differences in macrophages compared to L-BM (105, 107, 108), which strongly correlates with poor prognosis (109, 110). PD-1 to PD-L1 interaction between T lymphocytes and tumor cells leads to immune quiescence, impairing the immune system’s ability to recognize and attack tumor cells. The ligand-receptor complex elicits various reactions including suppression of IL-2 synthesis required for T cell survival, differentiation, and self-tolerance (109, 110) as well as activation of Src homology region 2 domain containing phosphatase-2 (SHP2), which dephosphorylates T-cell receptor-associated CD3 and ZAP70 signalosomes thereby inhibiting cytokine secretion and attenuating cytokine-triggered signaling (111).

One important regulator of PD-L1 is the tumor suppressor gene *p53*, which is mutated in approximately 50% of NSCLC cases (111). Aberrant *p53* expression associates with PD-L1 positivity, aggressive clinicopathologic features, and shorter survival in NSCLC (112, 113). PD-L1 expression is notably elevated *KRAS*-mutant and *ALK*-positive NSCLC, activating PI3K-AKT-mTOR and MEK-ERK signaling pathways implicated in metastatic spread and CNS tropism (114–118). Among *KRAS* mutations, the G12C subtype typically shows the highest rates of PD-L1 positivity (54%), compared to 46% for all *KRAS* mutations and 38% for the overall NSCLC population (119). Expression of the immune checkpoint PD-1 by cytological or histological analysis (120) has served a molecular biomarker of favorable response to immunotherapies, alone or in combination with standard of care or targeted therapies against the genetic drivers (121).

Like PD-L1, CTLA-4 also negatively regulates T-cell activation by binding to the co-stimulatory receptor CD28 on antigen-presenting cells and facilitating immunological tolerance (122). Therapeutic immune checkpoint inhibitor (ICI) antibodies prevent immune checkpoint interactions, thereby reversing immune cell avoidance and enhancing anti-tumor immunity (123). To date, the FDA has approved 6 ICIs consisting of 1) PD-1 inhibitors such as pembrolizumab, nivolumab, and cemiplimab, 2) PD-L1 inhibitors such as atezolizumab and durvalumab, and 3) a CTLA-4 inhibitor known as ipilimumab (102, 122, 124). ICI treatment indeed prolongs survival of L-BM patients, although prognosis becomes unfavorable with increasing L-BM lesion numbers and size (125–127).

While PD-L1 predicts ICI response and prognosis, evidence does not support a correlation between PD-L1 positivity and the risk of developing CNS metastases. ICIs benefit wildtype NSCLC (i.e. without genetic drivers) (121), and efficacy of ICI treatment of L-BM can be monitored through cerebrospinal fluid analysis for circulating PD-1+ T cells. In the context of the tumor cells, Lee and colleagues conducted a retrospective study of 270 patients in 2021 where PD-L1 positivity had similar two-year CNS progression rates compared to PD-L1-negative patients (26.3% vs. 28.4%, *p* = 0.944), and PD-L1 status did not affect CNS progression or overall survival in those with synchronous brain metastases (128). Another systematic review and meta-analysis of 230 patients performed by Tonse and colleagues in 2021 also reported PD-L1 discordance rates of approximately 20% between primary tumors and L-BM but did not identify PD-L1 as a determinant of CNS spread or prognosis (129).

The data demonstrating the efficiency of ICIs in NSCLC L-BM is limited, though studies have indicated significant survival advantages associated with ICIs. For instance, a pooled analysis of multiple trials (KEYNOTE-001, 010, 024, and 042) revealed that pembrolizumab extended overall survival (OS) compared to chemotherapy (13.4 months vs. 10.3 months, HR = 0.83) (102). Notably, in patients with PD-L1 expression ≥50%, the survival benefit was even greater (19.7 months vs. 9.7 months, HR = 0.67) (130). Similarly, a combined analysis of CheckMate-017 and 057 showed that nivolumab improved OS (7.6 months vs. 6.2 months, HR = 0.81) and the five-year OS rate (8% vs. 0%) compared to docetaxel (130). Cemiplimab, a recently approved anti-PD-1 agent, also showed enhanced progression-free survival (PFS) and OS in the BMs subgroup compared to platinum-based chemotherapy (130). Atezolizumab delayed onset of new BMs when compared to docetaxel, with median time to development of new BMs not reached at 9.3 months (HR = 0.38). The PACIFIC trial demonstrated that maintenance therapy with durvalumab following chemoradiotherapy significantly reduced the incidence of new BMs (6.5% vs. 11.8%) at five-year follow up (130). Collectively, these findings indicate that PD-L1 inhibitors offer significant survival advantages over traditional therapies. However, PD-L1 status does not appear to influence CNS metastatic spread, emphasizing the need for additional biomarkers in L-BM prediction.

## Limitations in patient-centered outcomes

While advances in molecularly targeted agents and immune therapies have markedly improved systemic and CNS disease control in NSCLC brain metastases, key gaps remain in assessing patient-centered outcomes. Many trials report on systemic toxicities and drug related adverse-events, yet the overall quality-of-life (QoL) and neurocognitive profile of these treatments remains inadequately characterized - critical aspects that require further research to ensure truly patient-centered care.

For example, the FLAURA2 trial has cited similar safety outcomes among patients with baseline CNS lesions and the overall study population. It reports a doubling in the frequency of grade 3 or higher adverse events (AEs) (64% versus 29%), serious AEs (37% versus 22%), and fatal AEs (6% versus 3%) in the combination arm compared to the osimertibib monotherapy arm (131). HERTHENA-Lung01 (phase II, patritumab deruxtecan) documented hematologic side effects such as thrombocytopenia, neutropenia, anemia, and leukopenia. Yet, despite safety reporting, details on neurotoxicity, neurocognitive decline, or patient-reported QoL metrics are often limited or entirely absent (132). Among the few exceptions, the CROWN and ALTA-1L trials both incorporated patient-reported outcomes data using validated cancer-related quality of life questionnaires such as the EORTC and QLQ-C30. The ALTA-1L trial demonstrated that brigatinib significantly delayed the time to deterioration of global health status (GHS)/quality of life (QoL) compared to crizotinib in patients with advanced *ALK*-positive NSCLC (26.74 mo vs. 8.31 mo) (133). Similarly, CROWN showed that lorlatinib maintained or improved QoL, functioning, and symptom burden, yet had a higher incidence of adverse neurocognitive effects - cognitive, mood, psychotic, and speech related (134). Additionally, CodeBreaK 200 utilized multiple instruments (EORTC QLQ-C30, EORTC QLQ-LC1330, FACT-G, PRO-CTCAE) to assess patient perception of symptom burden, supporting that sotorasib may be a more tolerable option than docetaxel for *KRAS*-mutant NSCLC given the lesser severity of pain, muscle and joint aches, as well as reduced interference with daily activities (135).

Despite these efforts, comprehensive assessment of QoL and neurocognitive considerations in L-BM patients remains largely overlooked in clinical research. Several factors contribute this. For one, most clinical trials prioritize hard clinical outcomes and on efficacy via objective, quantifiable endpoints like progression-free survival, overall survival, response rate, and duration of response (136). Subjective experiences such as memory complaints and psychological wellbeing present a high degree of heterogeneity throughout the disease process, progression, and experience (136). The distinct brain pathology across etiologies and varying response to the disease process makes difficult to draw valid statistical and clinical inferences and raises the risk of misinterpreting intervention efficacy, disease progression, or functional status in patients (136).

Moreover, DiRisio and colleagues report discrepancies in the assessment of health-related quality of life among patients with brain metastases due to variability in the tools and approaches used. Many patient-reported outcome measures (PROMs), such as EORTC QLQ-30 and FACT-G- which have been employed in trials such as ALTA-1L and CodeBreaK 200 - were originally developed for general cancer populations, and do not adequately address the unique neurocognitive challenges experienced by L-BM patients (137). These tools often focus on capturing functional symptoms over cognitive impairments, resulting in insufficient quality of PROM data reporting for L-BM patients undergoing diverse treatment modalities (137).

Matsui and colleagues highlight that neurocognitive decline in BM patients is associated with significant reductions in QoL including memory and executive function deficits caused by both the tumor and treatments like whole-brain radiation therapy (WBRT), on-radiation treatments (chemotherapy, immunotherapy, surgery), additional medications (steroids, anti-depressants), and disease progression (138). While these therapies may prolong overall survival, they also increase the risk of complications and treatment-related adverse events, underscoring the importance of including a more comprehensive evaluation of treatment impact and disease progression to improve patient-centered care in patients with brain tumors and metastases.

## Future directions

### Emerging CNS-directed EGFR-targeted combinations

The future of treatment likely lies in combinatorial therapy for patients with L-BM. For example, a trial of carboplatin and pemetrexed in combination with lazertinib, a newer third-generation EGFR inhibitor, is ongoing (139). Lazertinib is also being investigated in combination with amivantamab, a dual EGFR and cMET inhibitor [NCT05746481]. Other novel third-generation EGFR inhibitors under study include almonertinib, keynatinib, rezivertinib, and alflutinib [NCT04965090].

### Next-generation immunotherapy and bispecific checkpoint blockade

In the immunotherapy space, tiragolumab to the combination of carboplatin, pemetrexed, and atezolizumab in patients with NSCLC and untreated L-BMs (140). Tiragolumab, which targets TIGIT (T-cell immunoreceptor with immunoglobulin and immunoreceptor tyrosine-based inhibition motif domain) provides another option for immune checkpoint inhibition beyond PD-1/PD-L1 or CTLA-4 (141). TIGIT inhibits T-cell activation by inhibiting stimulatory immune signaling from CD226 (141, 142). Candonilimab is a bispecific antibody that targets both PD-1 and CTLA-4 which may be less toxic than traditional combinatorial therapies like ipilimumab and nivolumab and is being trialed alone and in combination with chemotherapy and bevacizumab in patients with NSCLC and untreated L-BM (143, 144).

### Antibody-drug conjugates and novel cytotoxic platforms

Chemotherapy treatment modalities are also evolving for better drug delivery. Patritumab deruxtecan, an antibody-drug conjugate (ADC), is currently being assessed for efficacy in patients with L-L-BM or leptomeningeal disease [NCT05477615][NCT04965090, NCT05746481] (140–142),. Recently approved for use against breast cancer, sacituzumab govitecan-hziy is also being trialed in NSCLC patients, including L-BM [NCT05812534].

### Targeting DNA damage response pathways in L-BM

Furthermore, novel agents such as berzosertib, an inhibitor of ATR (ataxia telangiectasia and Rad3-related protein kinase) shows promise by disrupting DNA damage repair pathways. [NCT05865990]. Because of this mechanism, it is believed that berzosertib may synergize with chemotherapy and/or radiation with at least one study combining the drug with whole brain radiation (84) [NCT05865990].

### Innovative CNS drug−delivery strategies

Therapeutic management of NSCLC brain metastases faces unique challenges due to the BBB, which restricts drug penetration and efficacy across all cancer types with CNS involvement. Advances in molecularly targeted therapies have introduced sequential generations of TKIs - such as osimertinib for EGFR, lorlatinib for ALK, and entrectinib for ROS1 – which exhibit enhanced BBB penetration and improved survival outcomes. However, CNS relapse remains common. Furthermore, resistance mechanisms - including mutation-dependent alterations in drug binding or increased ABC transporter activity - may further restrict drug efficacy at the BBB (145–147).

To address these challenges, several innovative strategies are under active investigation and early clinical translation. Approaches such as intranasal administration and intraventricular/intrathecal delivery can deliver peptides, proteins, and antibodies to the CNS within minutes while avoiding first-pass metabolism, enhancing therapeutic concentration at brain metastatic sites (148). Clinical and translational studies of intraventricular chemotherapy and immuno−/virotherapy show that delivering agents directly into the ventricles or CSF bypasses many BBB constraints and allows more targeted exposure of brain and leptomeningeal metastases to therapeutics (149, 150). Other modalities such as focused ultrasound with microbubbles can transiently disrupt the BBB, permitting higher drug entry into parenchyma, although long−term safety and standardization remain under investigation (151, 152). Emerging molecular “Trojan horse” and nanocarrier technologies exploit receptor-mediated transcytosis or transport systems such as transferrin receptor (TfR) and low−density lipoprotein receptor−related protein−1 (LRP1) to enhance CNS uptake of anticancer agents (153). In addition, pharmacological or genetic inhibition of efflux transporters such as P-glycoprotein represents another goal, though clinical efficacy remains to be demonstrated on a broad scale (154). Looking ahead, truly effective CNS disease management will likely depend on integrating these advanced drug delivery platforms with subtype-directed molecular therapies.

### Integrating patient-reported outcomes in future trials

As molecular therapies continue to expand the treatment landscape for NSCLC-BM patients, future trials must expand beyond traditional endpoints such as progression-free survival and response rate to integrate patient-centered outcomes as a core component of clinical design. This includes the use of brain-tumor specific PROMs, such as Functional Assessment of Cancer Therapy-Brain (FACT-Br), Brain Neoplasm Module (EORTC QLQ-BN20), and the BC-Brain Patient Reported Outcome Questionnaire to evaluate the physical, social, emotional, and functional wellbeing specifically for brain tumor patients (155, 156). The FDA’s 2024 core-patient reported outcomes document recognizes the strong scientific rationale of incorporating PROMs into the benefit/risk assessment in clinical trials, along with standardized efficacy assessments using overall survival and tumor measures, to fully capture patient perspectives (157).

In addition to PROMs, objective neuropsychological evaluations should also be integrated into clinical trials, particularly those involving CNS-penetrating agents, along with long-term follow-up protocols (137). Together, these strategies will allow researchers to understand the durability of patient-centered benefits or harms as QoL and cognitive changes emerge and evolve over the course of the treatment.

## Conclusions

Molecular biomarkers are central to guiding treatment for NSCLC and L-BM. Overall, studies agree that molecular variations in *KRAS, EGFR, ALK, PD-L1*, and *ROS1* correlate with risk of L-BM and targeting these changes improve outcomes for patients with L-BM. Current clinical trials are increasingly, focusing on combining targeted therapy, immunotherapy, and traditional chemotherapy to enhance survival, overcome resistance mechanisms, and improve CNS drug penetration – one of the principal challenges in L-BM management. Future progress will depend on deeper integration of genomic and transcriptomic profiling, including comparative analyses of brain and extracranial metastases, to refine risk stratification. Importantly, as treatment efficacy improves, maintaining and improving quality of life (QoL) must also remain a central goal to ensure that extended survival is accompanied by preserved wellbeing. Such efforts will allow ultimately pave the way for effective, personalized treatment regimens, improving both quality of life and survival for patients with brain metastases from NSCLC.
